# SRA inhibition improves antitumor potency of antigen-targeted chaperone vaccine

**DOI:** 10.3389/fimmu.2023.1118781

**Published:** 2023-01-30

**Authors:** Jie Qian, Xiaofei Yu, Zheng Liu, Jinyang Cai, Masoud H. Manjili, Hu Yang, Chunqing Guo, Xiang-Yang Wang

**Affiliations:** ^1^ Department of Human & Molecular Genetics, Virginia Commonwealth University School of Medicine, Richmond, VA, United States; ^2^ Department of Microbiology and Immunology, Virginia Commonwealth University School of Medicine, Richmond, VA, United States; ^3^ Massey Cancer Center, Virginia Commonwealth University School of Medicine, Richmond, VA, United States; ^4^ Linda and Bipin Doshi Department of Chemical and Biochemical Engineering, Missouri University of Science and Technology, Rolla, MO, United States; ^5^ Institute of Molecular Medicine, Virginia Commonwealth University School of Medicine, Richmond, VA, United States; ^6^ Hunter Holmes McGuire VA Medical Center, Richmond, VA, United States

**Keywords:** immunotherapy, vaccine, heat shock protein 110, scavenger receptor A (SRA), dendritic cell

## Abstract

We have previously demonstrated that scavenger receptor A (SRA) acts as an immunosuppressive regulator of dendritic cell (DC) function in activating antitumor T cells. Here we investigate the potential of inhibiting SRA activity to enhance DC-targeted chaperone vaccines including one that was recently evaluated in melanoma patients. We show that short hairpin RNA-mediated SRA silencing significantly enhances the immunogenicity of DCs that have captured chaperone vaccines designed to target melanoma (i.e., hsp110-gp100) and breast cancer (i.e., hsp110-HER/Neu-ICD). SRA downregulation results in heightened activation of antigen-specific T cells and increased CD8^+^ T cell-dependent tumor inhibition. Additionally, small interfering RNA (siRNA) complexed with the biodegradable, biocompatible chitosan as a carrier can efficiently reduce SRA expression on CD11c^+^ DCs *in vitro* and *in vivo*. Our proof-of-concept study shows that direct administration of the chitosan-siRNA complex to mice promotes chaperone vaccine-elicited cytotoxic T lymphocyte (CTL) response, culminating in improved eradication of experimental melanoma metastases. Targeting SRA with this chitosan-siRNA regimen combined with the chaperone vaccine also leads to reprogramming of the tumor environment, indicated by elevation of the cytokine genes (i.e., *ifng*, *il12*) known to skew Th1-like cellular immunity and increased tumor infiltration by IFN-γ^+^CD8^+^ CTLs as well as IL-12^+^CD11c^+^ DCs. Given the promising antitumor activity and safety profile of chaperone vaccine in cancer patients, further optimization of the chitosan-siRNA formulation to potentially broaden the immunotherapeutic benefits of chaperone vaccine is warranted.

## Introduction

It has been well established that heat shock proteins (HSPs) with their superior chaperoning property can be utilized to carry and present tumor-associated antigens for effectively inducing antitumor immune responses (1–3). Building on this unique feature, we have developed synthetic chaperone vaccines by complexing large HSPs (e.g., hsp110, grp170) non-covalently with clinically relevant tumor protein antigens (e.g., gp100, HER-2/Neu) and demonstrated their potent immunotherapeutic activities involving CD8^+^ cytotoxic T lymphocytes (CTLs) in multiple preclinical cancer models (4–8). Mechanistic studies reveal that chaperone vaccines preferentially target specialized antigen-presenting cells (APCs), particularly dendritic cells (DCs) (9), which can be attributed to the presence of HSP-binding receptors facilitating the efficient capture and cross-presentation of the HSP-antigen complexes (2, 10). The recombinant human hsp110-gp100 chaperone vaccine was recently tested in a phase Ib dose-escalation trial (NCT01744171), which showed minimal toxicity, measurable antitumor efficacy, and capacity to activate CD8^+^ T cells in patients with advanced, pretreated melanoma (11). These preclinical and clinical evidence strongly support the therapeutic applications using these antigen-targeted chaperone vaccines to generate and expand tumor-reactive T cells for cancer eradication.

Scavenger receptor A (SRA, also known as CD204) is an innate pattern recognition receptor (PRR) primarily expressed on the cells of myeloid origin (e.g., DCs, macrophages) and displays pleiotropic biological as well as pathological activities, possibly due to its ability to bind a broad spectrum of ligands or macromolecules (12–15). We have shown that SRA acts as an important regulator capable of dampening the immunostimulatory function of DCs in promoting T cell-mediated antitumor immunity (16–21), suggesting that targeted inhibition of this immunosuppressor may lead to improved DC functionality for T cell priming and DC-targeted cancer immunotherapies (22). SRA has also been considered as a phenotypic maker for alternatively activated or M2-like macrophages and is involved in the functional regulation of tumor-associated macrophage for cancer promotion (23, 24). Given that the immunosuppressive activity of SRA can similarly attenuate antitumor immune responses augmented by chaperone vaccines (e.g., hsp110-gp100 complex), which require the internalization and processing by DCs prior to their activation of CTLs (16, 25), a question was raised on whether strategic inhibition of SRA can improve T cell-mediated antitumor immunity mobilized by the chaperone vaccines.

In this study, we have examined experimental approaches involving short hairpin RNA (shRNA) and small interfering RNA (siRNA) to achieve downregulation of SRA on DCs. We demonstrate that reduction of SRA greatly enhances the immunogenicity of DCs that have captured the chaperone vaccines and consequent antitumor immune responses. Furthermore, we show that administration of SRA siRNA carried by biocompatible and biodegradable chitosan (26, 27) can effectively decrease SRA expression on DCs *in vivo* and potentiate immunotherapeutic efficacy of chaperone vaccines against established cancer metastases.

## Materials and methods

### Mice and cell lines

C57BL/6 mice, Pmel transgenic mice carrying TCR gene specific for the mouse homolog (Pmel-17) of human gp100 (28), parental FVB mice and FVBN202 transgenic mice were purchased from the Jackson Laboratory (Bar Harbor, ME). Melanoma cell line B16-gp100 (8), mammary tumor MMC line (4), and DC1.2 DC line were maintained in DMEM media supplemented with 10% heat-inactivated FBS (Life Technologies, Grand Island, NY), 2 mM L-glutamine, 100 U/ml penicillin, and 100 mg/ml streptomycin. All experimental procedures were conducted according to the protocols approved by the Virginia Commonwealth University Institutional Animal Care and Use Committee (Richmond, VA).

### Reagents and antibodies

Recombinant proteins including human hsp110, gp100 and intracellular domain (ICD) of HER-2/Neu were expressed in a BacPAK™ baculovirous expression system (BD Biosciences Clontech, Palo Alto, CA) as we previously described (4, 5, 29). All glassware was depyrogenated for 4 h at 250 °C to avoid or reduce endotoxin contamination. Endotoxin levels in the recombinant protein preparations are approximately 10-15 EU/mg protein. H-2D^b^ restricted gp100_25-33_ (KVPRNQDWL) peptide was purchased from AnaSpec (Fremont, CA). Chitosan G213 were purchased from Novamatrix (Lancaster, PA). siRNA for SRA (#1 siRNA GGAAAUGAGAUUUACAAUUTT; #2 siRNA GACUUAAUGAUAUUCUUCUTT; #3 siRNA CAUCUCAAGGUCCUAUGGATT; #4 siRNA AUUUGACGCACGUUCAAUGTT) were synthesized by Invitrogen (Waltham, MA). Anti-SRA polyclonal antibodies for immunoblotting and monoclonal antibodies (2F8) for flow cytometry analysis were purchased from R&D systems (Minneapolis, MN) and AbD Serotec (Raleigh, NC), respectively. Mouse monoclonal antibodies to CD4 (GK1.5), CD8a (53-6.7), IFN-γ (XMG1.2), CD90.1 (OX-7), CD11c (HL3), isotype control rat IgG2b (RTK4530), and IgG1 (RTK2071) were purchased from BioLegend (San Diego, CA).

### Downregulation of SRA by genetic silencing

Lentiviruses encoding mouse SRA shRNA or scramble shRNA were packaged using Phoenix cells co-transfected with pLKO.1 construct and pMD.G and pCMVΔR8.91 as we previously described (20). Bone marrow-derived DCs (BMDCs) were generated from bone marrow cells in the presence of mouse GM-CSF from PeproTech (Rocky Hill, NJ) (16). Day 6 BMDCs were infected with shRNA-encoding lentivirus in the presence of 4 μg/ml polybrene and 20 ng/ml GM-CSF, and collected 48 h later for analyses. Preparation of chitosan-siRNA complex was performed as described (30, 31). Briefly, chitosan was dissolved in sodium acetate buffer (0.2 mol/l NaAc, pH 4.5) to obtain a 3.2 mg/ml solution and then adjusted to pH 5.5. Chitosan and siRNA were thoroughly mixed at N:P of 60 and left at room temperature for 1 h. Cells were incubated with the chitosan-siRNA complex (200 nM) in 500 μl FBS-free medium for 4 h, followed by adding 500 μl medium with 20% FBS and culturing for additional 72 h. For *in vivo* treatment, each mouse received 4 μg chitosan-siRNA complex *i.p.* in 200 μl sterile PBS.

### Expansion of ICD-specific T cells

The ICD-reactive CD8^+^ T cells were generated as we previously described (32). FVB mice were inoculated with 5 × 10^6^ HER-2/Neu-positive MMC cells and lymph node cells harvested after three weeks. Cells (10^6^ cells/mL) were cultured in complete RPMI1640 medium containing 15% FBS with bryostatin-1 (5 nM) and ionomycin (1 μM) plus IL-2 (80 U/mL) for 16 h. Cells were washed and cultured at 10^6^ cells/mL in complete medium with IL-2 (40 U/mL) and media was changed every other day for a total of 5 days. T cells were isolated by centrifugation using Percoll solution from Sigma-Aldrich (Saint Louis, MO).

### *In vitro* priming of T cells

For preparation of chaperone vaccines, recombinant hsp110 protein and gp100 protein or ICD protein (1:1 molar ratio) were incubated at 50 °C for 30 min, followed by incubation at 37 °C for 30 min as previously described (5). BMDCs were incubated with the chaperone-antigen complexes (30 μg/mL) for 3 h, and co-cultured with 5×10^4^ Pmel cells or ICD-specific T cells in 200 μl RPMI1640 medium in a round-bottom 96-well microtiter plate. Cells were cultured for 60 h and pulsed with ^3^H-thymidine (0.5 μci/well) during the last 16 h of culture period, followed by ^3^H-TdR incorporation assays for T cell proliferation. IFN-γ production in the culture supernatant was determined using ELISA kits from eBioscience (San Diego, CA). For some experiments, T cells were labeled with 2.5 μM CFSE before co-culture with DCs. T cell proliferation was measured by flow cytometry analysis (17).

### Adoptive cell transfer

Antigen-specific CD8^+^ T cells (5×10^6^) were transferred into recipient mice *via* tail vein injection. Mice were immunized *s.c.* next day with chaperone vaccine-loaded DCs. In some cases, mice received *i.p.* chitosan-siRNA complex combined with chaperone vaccines. Spleen or lymph nodes were recovered 4 days later and subjected to intracellular cytokine staining and flow cytometry analysis (25).

### *Ex vivo* assays for T cells

Mice were immunized twice at 2-week intervals with DCs pulsed with chaperone vaccines *s.c.* or with the chitosan-siRNA complex plus hsp110-gp100 vaccine. For intracellular IFN-γ staining, cells were treated with brefeldin A (5 μg/mL, BD GolgiPlug; BD Biosciences) for 3 h at 37 °C. In some cases, cells were stimulated with phorbol-12-myristate-13-acetate (PMA, 50 ng/mL) plus ionomycin (1 μg/mL). Cells were stained with anti-CD8 or CD90.1 antibodies, followed by fixation, permeabilization and staining with anti-IFN-γ antibodies (BD Biosciences). Cells were analyzed by gating on CD8^+^ or CD8^+^CD90.1^+^ T cells. To examine the frequency of IFN-γ-producing T cells, cells were stimulated with gp100_25–33_ peptide and subjected to ELISPOT analysis (25). An *in vivo* CTL assay was performed to determine the cytolytic activity of gp100-specific CTLs as we previously described (17).

### Tumor therapy study

Mice were established with tumors by injecting 2 × 10^5^ B16-gp100 cells *s.c.* on day 0, followed by immunization with differentially treated DCs on days 4, 7, and 10. Tumor growth was monitored by measuring tumor sizes with a digital caliper. The tumor volume is calculated using the formula V = (The shortest diameter^2^ × the longest diameter)/2. In some experiments, mice were depleted of CD4^+^ or CD8^+^ T cells using anti-CD4 (Clone GK1.5) or anti-CD8 (Clone 2.43) antibodies, respectively (16). For analysis of tumor-infiltrating immune cells, tumors were digested with collagenase D (10 μg/mL) and DNase I (100 μg/mL) for 1 h at 37˚C. Single cell suspensions were prepared for flow cytometry analysis as described (33).

### Treatment of experimental lung metastases

Mice were established with lung metastases by *i.v.* inoculation of 4 ×10^5^ B16-gp100 cells in 200 μL of PBS. Mice were treated *i.p.* with the chitosan-siRNA complex in combination with hsp110-gp100 chaperone vaccine on days 4, 7, 10, and 13. Mice were euthanized on day 16 and the lungs were subjected to hematoxylin and eosin staining.

### Statistical analysis

Comparisons between two groups were performed using Student’s *t* test. Comparisons between multiple groups were carried out using ANOVA test. Log-Rank (Mantel-Cox) test was performed to compare the survival curves. A value of *p* < 0.05 is considered to be statistically significant.

## Results

### SRA silencing increases the immunogenicity of DCs upon interaction with chaperone vaccines

**Figure 1:**
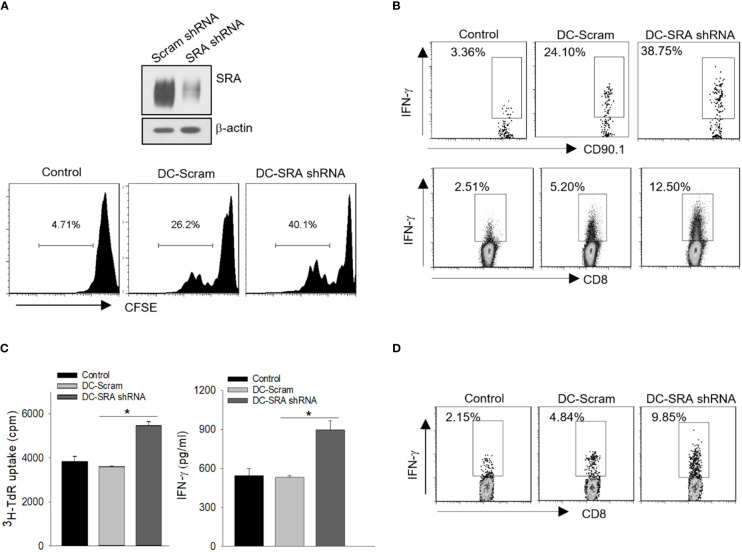
SRA silencing in DCs enhances activation of antigen-specific CD8^+^ T cells. **(A)** DCs were treated with a lentivirus encoding short hairpin RNA (shRNA) for SRA or scramble (Scram) control followed by immunoblotting analysis of SRA expression (top). These DCs pulsed with hsp110-gp100 complex vaccine were then co-cultured with CFSE-labeled, gp100-specific Pmel T cells at a ratio of 1:5. T cell proliferation was examined using CFSE dilution assay (bottom). **(B)** C57BL/6 mice were adoptively transferred with naïve Pmel CD90.1^+^CD8^+^ cells on day 0 and immunized on day 1 with SRA-silenced DCs that had been loaded with the complex vaccine. Lymph node cells were subjected to intracellular cytokine staining on day 5 for IFN-γ expression on CD90.1^+^ cells or CD8^+^ T cells. **(C)** SRA or scramble shRNA-treated DCs were pulsed with hsp110-ICD complex vaccine, and were co-cultured with ICD-reactive T cells for 3 days. ^3^H-thymidine uptake assay for cell proliferation and ELISA analysis of IFN-γ production in culture medium were performed. **(D)** FVBN202 transgenic mice were transferred with CFSE-labeled ICD-reactive T cells on day 0, followed by immunization on day 1 with SRA-silenced DCs or scramble shRNA treated controls pulsed with hsp110-ICD complex vaccine. Lymph node cells were examined for IFN-γ-producing CFSE^+^CD8^+^ cells on day 5. The results are representative of three independent experiments. *p < 0.05.

We first performed shRNA-based gene silencing to examine whether targeted inhibition of SRA enhances the immunostimulatory activity of DCs following capture of the hsp110-gp100 chaperone vaccine, which has been tested in mouse melanoma model (5, 8) and patients with advanced melanoma (11). A lentivirus encoding shRNA for SRA or scramble control was used to downregulate SRA as we previously described (**Figure 1A**) (20, 25). The shRNA-treated DCs were pulsed with the hsp110-gp100 vaccine prior to co-culture with CFSE-labeled, gp100_25-33_-specific CD8^+^ T cells derived from Pmel transgenic mice (28). SRA-silenced DCs were more efficient than mock-treated DCs in stimulating the proliferation of Pmel cells (**Figure 1A**), as shown in CFSE dilution assays (34). To determine the effect of SRA silencing on *in vivo* priming of naïve CD8^+^ T cells by these DCs, we adoptively transferred gp100_25-33_-specific CD90.1^+^ Pmel cells to mice, followed by immunization with DCs that had been loaded with the hsp110-gp100 vaccine. It was evident that SRA-silenced DCs were more immunogenic compared to those DCs treated with scramble shRNA, indicated by a significant increase in IFN-γ-producing CD90.1^+^ Pmel cells or CD8^+^ T cells based on intracellular cytokine staining and flow cytometry analysis (**Figure 1B**).

To validate our results, we examined the immunostimulatory activity of SRA-silenced DCs after incubation with the hsp110-ICD chaperone vaccine that was designed to target breast cancer (4, 6). The SRA-silenced DCs that were carrying the hsp110-ICD complex resulted in enhanced proliferation and activation of the ICD-reactive T cells (**Figure 1C**). *In vivo* T cell priming assays showed that SRA silencing conferred DCs increased functionality in stimulating adoptively transferred ICD-reactive CD8^+^ T cells (**Figure 1D**), suggesting that targeted inhibition of SRA may be used to increase the immunotherapeutic potency of chaperone-based cancer vaccines.

### SRA-silencing enhances antitumor immunity induced by chaperone vaccine carrying DCs

**Figure 2:**
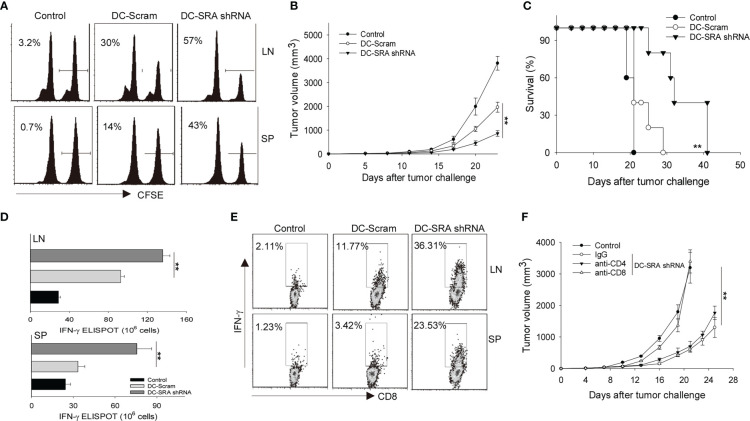
SRA-silencing potentiates antitumor immunity induced by DCs carrying chaperone vaccine. **(A)** Mice received gp100_25–33_ pulsed, CFSE-labeled splenocytes, followed by immunization with SRA-silenced DCs or scramble shRNA-treated DCs that were carrying the hsp110-gp100 complex vaccine. The cytolytic activity of gp100-specific T cells was examined by *in vivo* CTL assay. Percentage of target killing is indicated by the numbers in parentheses. **(B, C)**. Mice (n=5) established with B16-gp100 tumors were immunized with indicated DCs carrying the hsp110-gp100 complex vaccine. Tumor progression **(B)** and survival of mice **(C)** were followed. **(D)** Splenocytes (SP) and tumor-draining lymph node (LN) cells from treated mice were stimulated with gp100_25–33_ and subjected to ELISPOT assay for the frequencies of antigen-specific CD8^+^ T cells. **(E)** Splenocytes and tumor-draining LN cells were also examined by intracellular IFN-γ staining analysis for T cell activation. **(F)** B16-gp100 tumor-bearing mice (n=5) were depleted of CD4^+^ or CD8^+^ T cells using antibodies prior to immunization with DC-SRA shRNA as indicated. Untreated tumor bearing mice were used as controls. Data are representative of three independent experiments with similar results. **p < 0.01.

We next carried out *in vivo* CTL assays to assess the cytotoxic activity of gp100-specific CD8^+^ CTLs following immunization with SRA-silenced DCs presenting the hsp110-gp100 chaperone vaccine. Mice were injected *i.v.* with 50% CFSE^high^ splenocytes cells pulsed with gp100_25-33_ peptides mixed with 50% CFSE^low^ cells without peptide treatment as controls (17). Flow cytometry analysis showed that immunization with SRA-silenced DCs resulted in a higher efficiency of gp100_25-33_-specific killing than did those mock-treated DCs (**Figure 2A**). To further examine the therapeutic effect of SRA silencing in DCs, we treated B16-gp100 melanoma bearing mice with the hsp110-gp100 vaccine-loaded DCs that have received either SRA shRNA or scramble shRNA. Immunotherapy with the SRA-silenced DCs led to significantly improved tumor control (**Figure 2B**) and prolonged survival of mice (**Figure 2C**) compared to their mock-treated counterparts. We also assessed the immune status in the tumor-draining lymph nodes and spleen to understand the basis of enhanced antitumor activity of SRA-silenced DCs. It was shown that mice receiving hsp110-gp100 vaccine pulsed, SRA-silenced DCs developed a higher level of gp100_25-33_-specific IFN-γ^+^CD8^+^ T cells, assayed by using ELISPOT (**Figure 2D**) or intracellular cytokine staining (**Figure 2E**).

To determine the relative contributions of T cell subsets to the improved tumor inhibition by SRA-silenced DCs, we removed either CD4^+^ or CD8^+^ T cells from mice using respective depleting antibodies. We showed that the absence of CD8^+^ T cells abrogated the antitumor activity (**Figure 2F**), supporting a critical role of antitumor CTLs mobilized by SRA-silenced DCs.

### SRA-silenced DCs carrying chaperone vaccine reprograms the tumor environment

**Figure 3:**
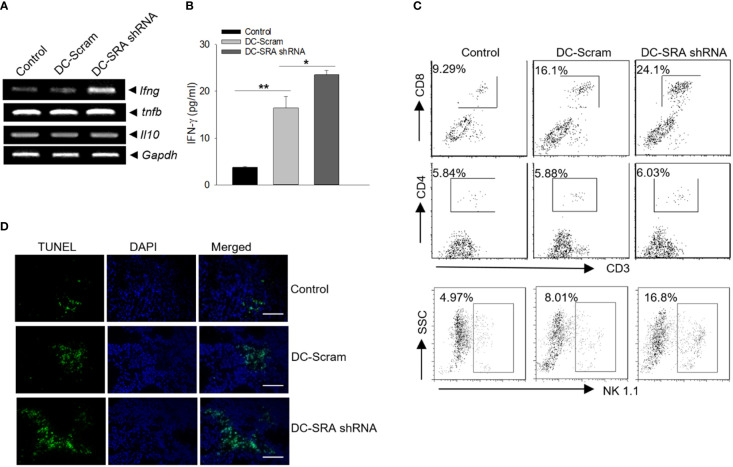
Improved immune activation in the tumor sites following immunotherapy with SRA-silenced DCs carrying the chaperone vaccine. **(A)** Mice with established B16-gp100 tumors were immunized with SRA-silenced DCs loaded with the hsp100-gp100 complex vaccine, followed by PCR analyses of immune-related genes in tumor tissues. **(B)** The levels of IFN-γ was also examined using ELISA. **(C)** Tumor infiltrating by CD4^+^ or CD8^+^ T cells as well as NK cells was analyzed using flow cytometry. **(D)** Tumor tissues from treated mice were subjected to TUNEL assays for tumor cell death. Bar = 50 um. The results are representative of three independent experiments. *p < 0.05; **p < 0.01.

We investigated the potential alterations of immune status in the tumor sites since tumor-immune interplay is an important determinant of immunotherapeutic outcomes. We found that there was transcriptional upregulation of the gene *ifng* but not *tnfb* (also known as lymphotoxin-α) or *il10* in the tumor tissues from mice receiving SRA-silenced DCs carrying the hsp110-gp100 vaccine (**Figure 3A**). The increased level of the cytokine IFN-γ, a signature of Th1 dominant immunity critical for tumor suppression and elimination, was further confirmed by tissue ELISA analysis (**Figure 3B**). Consistent with this observation, there were more CD8^+^ T cells as well as natural killer (NK) cells, which are well recognized for their IFN-γ-producing capacity, in the tumors of mice receiving SRA-silenced DCs compared to those receiving mock-treated cells (**Figure 3C**). Additionally, this enhanced immune activation in the tumor sites correlated with increased cancer cell death, as shown by TUNEL assays (**Figure 3D**).

### SRA knockdown by the chitosan-SRA siRNA complex *in vitro* and *in vivo*

**Figure 4:**
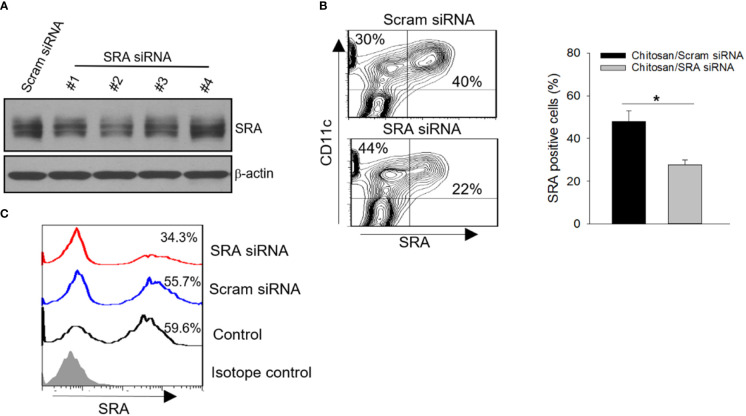
SRA knockdown by chitosan-SRA siRNA complex *in vitro* and *in vivo*. **(A)** Screening of small-interfering RNAs (siRNAs) in complex with chitosan for effective downregulation of SRA expression in DC1.2 cell line. **(B)** Validation of SRA downregulation in bone marrow-derived DCs following treatment with the chitosan-SRA siRNA nanoparticle complex. Representative flow cytometry results and quantification of SRA-expressing DCs are shown. **(C)** C57BL/6 mice were injected with chitosan-SRA siRNA complex (5 μg/mouse) on days 0 and 2. Cells in lavage fluid were collected on day 5 and analyzed for SRA expression on CD11c^+^ cells. *p < 0.05.

We next examined the possibility of targeting SRA *in vivo* to improve chaperone vaccine-induced antitumor immune response. Given that biocompatible and biodegradable chitosan exhibits unique features in the formulation of siRNA and can readily form nanoparticles for efficient delivery (26, 27), we chose chitosan for complexing with siRNA to achieve targeted inhibition of SRA. Based on our pilot study, N:P ratio of 60 was used to prepare the chitosan-siRNA complex (**Supplementary Figure 1A**). We also showed that DCs can efficiently internalize chitosan due to their endocytic property (**Supplementary Figure 1B**). Using DC1.2 cell line, we first compared SRA siRNAs with different targeting sequences for their knockdown efficiency when complexing with chitosan. siRNA #2 with targeting sequence GACUUAAUGAUAUUCUUCUTT was selected based on its activity in downregulating SRA expression (**Figure 4A**). Consistent with other studies, the chitosan readily formed into nanoparticles with sizes of approximately 119 nm after complexing with SRA siRNA (data not shown). The knockdown effect of the chitosan-SRA siRNA complex was also confirmed using primary DCs-derived from bone marrow cells (**Figure 4B**). To test its ability to reduce SRA expression on DCs *in vivo*, we injected the chitosan-SRA siRNA complex into the peritoneal cavity. Flow cytometry analysis showed that the chitosan-siRNA formulation was effective in downregulating SRA levels on CD11c^+^ cells within the lavage fluid (**Figure 4C**).

### The chitosan-SRA siRNA complex augments chaperone vaccine-elicited CTL response

**Figure 5:**
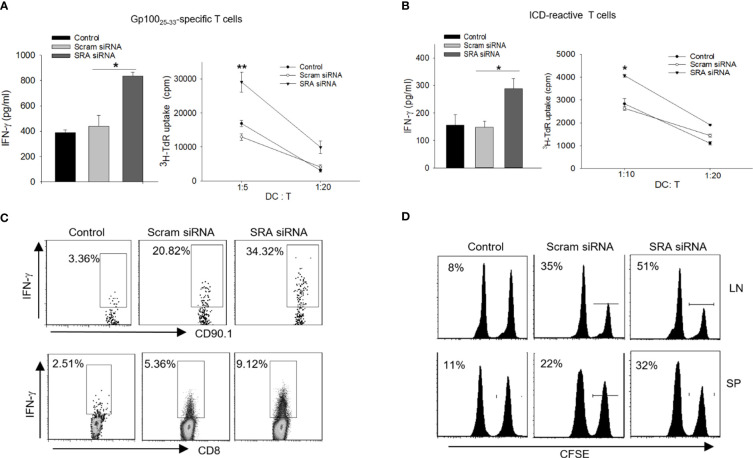
Chitosan-SRA siRNA complex augment chaperone vaccine-induced antigen-specific CTL response. A-B. DCs were treated with SRA or Scram siRNA complexed with chitosan, followed by pulsing with hsp110-gp100 complex **(A)** or hsp110-ICD complex **(B)** for 5 h. DCs were then co-cultured with gp100 or ICD-specific T cells, respectively. ELISA analysis of IFN-γ production and ^3^H thymidine uptake assays for T cell proliferation were performed. **(C)** C57BL/6 mice were adoptively transferred with naive Pmel cells on day 0 and immunized on day 1 with chitosan-SRA siRNA complex together with chaperone vaccine. Lymph node cells were analyzed on day 5 for gp100_25-33_-reactive, IFN-γ-expressing CD8^+^ or CD90.1^+^ T cells using intracellular cytokine staining and flow cytometry. **(D)** *In vivo* CTL assays were performed to assess the cytolytic activity of gp100-specific CD8^+^ T cells against gp100_25–33_-pulsed, CFSE-labeled splenocytes following immunotherapy. Percentage of target killing is indicated by the numbers in parentheses. The results are representative of three independent experiments. *p < 0.05; **p < 0.01.

We first examined the potential effect of chitosan on DCs and showed that treatment with chitosan by itself has little effect on the maturation/activation of DCs (**Supplementary Figure 1C**) or their antigen-presenting activity (**Supplementary Figure 1D**). We next asked the question whether this chitosan-SRA siRNA complex could enhance the immunogenicity of DCs during the processing of the chaperone vaccines. To approach this, DCs were treated with either chitosan-SRA siRNA or chitosan-scramble siRNA, followed by incubation with the hsp110-gp100 chaperone vaccine and co-culture with gp100_25-33_-specific Pmel cells. We showed that DCs treated with the chitosan-SRA siRNA complex were more effective than similarly treated control DCs in stimulating IFN-γ production as well as proliferation of gp100-specific T cells, measured by using ELISA and ^3^H-thymidine incorporation assays, respectively (**Figure 5A**). The similar results were obtained when DCs treated with the chitosan-SRA siRNA were co-cultured with ICD-reactive T cells (**Figure 5B**).

We further evaluated the immunostimulatory activity of the chitosan-SRA siRNA complex in mice receiving chaperone vaccine. Mice were first adoptively transferred with CD90.1^+^ Pmel cells, followed by *i.v.* administration of the hsp110-gp100 vaccine together with SRA siRNA or scramble siRNA complexed with chitosan. Intracellular cytokine staining analysis revealed that the chitosan-SRA siRNA complex significantly increased the frequency of IFN-γ-producing CD90.1^+^ cells or CD8^+^ T cells (**Figure 5C**), suggesting elevated activation of gp100_25-33_-specific CD8^+^ T cells. In support of this finding, *in vivo* CTL assays showed that mice receiving the chitosan-SRA siRNA complex plus the hsp110-gp100 vaccine exhibited improved ability to eliminate gp100_25-33_-positive targets (**Figure 5D**), supporting the feasibility of chitosan-based SRA siRNA delivery for boosting an antigen-specific CTL response.

### Targeting SRA with the chitosan-siRNA complex potentiates chaperone vaccine-induced antitumor immunity

**Figure 6:**
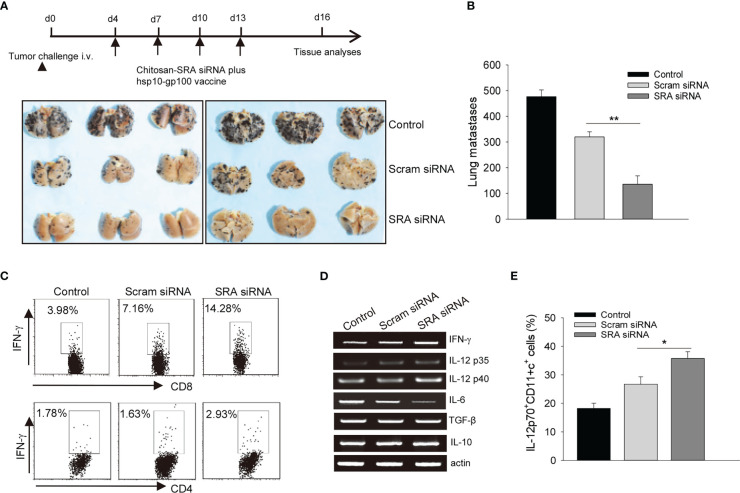
Targeting SRA using chitosan-siRNA complex augments chaperone vaccine-induced antitumor immunity. **(A, B)**. C57BL/6 mice (n=5) established with experimental lung metastases were treated with chitosan-SRA siRNA and hsp100-gp100 chaperone vaccine as described in the scheme of experimental procedure. Gross images of lungs **(A)** and the number of metastatic nodules **(B)** are shown. **(C)** Tumor-infiltrating cells were analyzed for IFN-γ-expressing CD8^+^ or CD4^+^ T cells. **(D)** Tumor tissues from treated mice were examined for immune-related gene expression as indicated. **(E)** Flow cytometry analysis of the frequency of IL-12p70^+^CD11c^+^ cells. The data are representative of three independent experiments with similar results. *p < 0.05; **p < 0.01.

Lastly, we evaluated the potential application of targeting SRA with the chitosan-SRA siRNA complex to improve the therapeutic outcomes of chaperone vaccines. C57BL/6 mice established with B16 melanoma-derived experimental lung metastases were treated with the hsp100-gp100 vaccine in combination with the chitosan-SRA siRNA or chitosan-scramble siRNA complex. We showed that the chitosan-SRA siRNA treatment profoundly reduced the number of metastatic nodules in mice receiving the hsp110-gp100 vaccine (**Figure 6A, B**). This enhanced eradication of metastases was associated with increased tumor infiltration by CD8^+^ and CD4^+^ T cells expressing IFN-γ (**Figure 6C**). PCR analysis of tumor tissues showed that targeted inhibition of SRA appeared to upregulate the cytokine genes *ifng*, *il12p40* and *il12p35* (**Figure 6D**), which are crucial for Th1-skewed antitumor immunity. Consistent with this result, the flow cytometry analysis further showed that the frequency of tumor-infiltrating IL-12p70^+^CD11c^+^ cells was significantly increased in mice receiving the chitosan-SRA siRNA compared to those treated with chitosan-scramble siRNA (**Figure 6E**), suggesting enhanced T cell-mediated antitumor immunity by targeted SRA inhibition involves improved functionality of DCs *in vivo*. Additionally, analysis of the immunization site (i.e., peritoneal cavity) showed that administration of the chitosan-SRA siRNA complex combined with the chaperone vaccine increased the recruitment of CD11c^+^ cells and their IL-12 expression (**Supplementary Figure 2**). In addition to increased activation of CTLs, we found that inhibition of SRA in the setting of immunization with chaperone vaccine resulted in elevation of antibodies to melanoma antigen gp100 (**Supplementary Figure 3**). We also evaluated the potential toxicity of the chitosan-SRA siRNA complex by examining the major organs (e.g., liver, kidney, spleen, lung) from mice that had been treated with or without the hsp110-gp100 vaccine. There were no detectable pathologic changes in these organs (data not shown), suggesting the chitosan-SRA siRNA formulation does cause any measurable side effects in mice.

## Discussion

The recombinant chaperone vaccine (e.g., hsp110-gp100) that is built on the superior antigen-carrying and delivering property of the evolutionarily conservative HSPs can efficiently generate antitumor immune responses in preclinical cancer models (5, 8). This melanoma antigen gp100-targeted vaccine was recently evaluated in patients with pretreated, unresectable stage IIIB/C/IV melanoma and displayed encouraging clinical tumor response, activation of gp100-specific CD8^+^ T cells as well as an excellent safety profile (11). In this study, we demonstrate that targeted inhibition of an immunosuppressive PRR, i.e., SRA, results in enhanced activation of antigen-specific CTLs and tumor eradication in the setting of immunotherapy with chaperone vaccines. Our findings provide experimental evidence supporting the feasibility of coupling chaperone vaccine with inhibition of SRA for improved cancer immunotherapy.

The immunological basis of chaperone vaccine involves the recognition and endocytosis of HSP-antigen complex preferentially and highly efficiently by the immune sentinel cells such as DCs (9). Our previous work uncovered an SRA-centered immunoregulatory pathway, which antagonizes the functional activation of DCs and consequent T cell priming induced by DC-targeted immunotherapies including chaperone vaccines (16, 17, 19–21, 25). Here we further elucidate that genetic silencing of SRA using shRNA increased the immunogenicity of DCs that have captured the hsp110-gp100 vaccine, assessed by DC-mediated activation of gp100_25-33_-specific T cells *in vitro* and *in vivo*, acquisition of cytolytic activity by T cells, growth inhibition of established melanoma, and prolonged mouse survival following DC immunization. The immunostimulatory effect of shRNA-mediated SRA downregulation on DC functionality has also been verified using chaperone vaccine targeting the breast cancer antigen HER-2/Neu, suggesting that the approaches to SRA inhibition may broadly enhance the antitumor potency of synthetic chaperone vaccines in the treatment of multiple disease indications.

Chitosan has been widely used for formulating nucleic acid-based therapeutics including siRNA due to its cationic nature, low toxicity, biodegradability and biocompatibility (26, 27), which are attractive drug delivery cargo compared to other viral or non-viral delivery vehicles. To achieve targeted inhibition of SRA *in vivo* to promote chaperone vaccine-elicited immune activation, we have tested the feasibility of combining the chitosan potential with the versatility of siRNA to downregulate SRA activity for an enhanced antitumor immune response in the setting of chaperone vaccine treatment. Consistent with other studies, the unique features of chitosan permit an efficient complex formation with SRA siRNA into nanoparticles. Furthermore, this chitosan-SRA siRNA complex is effective in reducing SRA expression in DCs *in vitro* and *in vivo* concomitantly with heightened DC functionality, which is supported by increased IFN-γ production and cytolytic activity of antigen-specific CTLs based on multiple complementary immune readouts, including ^3^H-thymidine uptake, ELISA or ELISPOT, intracellular cytokine staining, and *in vivo* CTL assays. Importantly, our proof-of-concept study has shown that administration of the chitosan-SRA siRNA in combination with chaperone vaccine profoundly reduces experimental lung metastases of melanoma compared to vaccination alone in mice.

In support of the robust antitumor response, the chitosan-SRA siRNA included in our combination immunotherapy regimen appears to help reprogram the immunologically ‘*cold*’ tumor microenvironment by recruiting more IFN-γ-producing CD8^+^ CTLs and IL-12^+^CD11c^+^ APCs, which are crucial for overcoming the immunosuppressive barriers in the tumor sites to achieve improved therapeutic outcomes. Since human gp100-specific CD8^+^ T cells were greater in the clinically responding patients with advanced melanoma in the hsp110-gp100 vaccine trial (11), our data suggest that the chitosan-based delivery of RNA-interfering therapeutic may be a promising approach to be exploited for promoting antitumor efficacy of chaperone vaccines in future clinical trials. However, additional studies in chitosan chemical modifications and formulation improvements are required to further optimize payload as well as targeting specificity and efficiency.

Another intriguing observation is that administration of the chitosan-SRA siRNA together with chaperone vaccine leads to more efficient induction of antibodies to the melanoma antigen gp100. A pattern of humoral cell responses to vaccination was not observed in patients receiving the hsp110-gp100 chaperone vaccine (11). There is also no significant difference in the levels of gp100 antibodies in wild-type or SRA deficient mice after immunization with the hsp110-gp100 chaperone vaccine (25). It is possible that the chitosan-SRA siRNA administrated combined with the hsp110-gp100 vaccine may facilitate a humoral response to the antigen target carried by the chaperone molecule, despite the recent reports showing that the chitosan nanoparticles as antigen vehicle trigger activation of antigen-specific CD8^+^ T cells (35). Additional studies are necessary to address whether the chitosan may be used to deliver both siRNA therapeutic and protein-based chaperone vaccine, and whether this antibody responses to gp100 also contributes to tumor suppression resulting from the combinatorial immunotherapy.

In conclusion, using shRNA and siRNA-based gene targeting approaches we provide additional evidence underscoring the immunosuppressive function of SRA in DCs that antagonizes chaperone vaccine-induced antitumor immunity. Our study supports the feasibility of using the chitosan-siRNA formulation to counteract the immunoregulatory action of SRA to improve antitumor outcomes in the context of chaperone vaccine-based immunotherapy. The *de nova* generation and expansion of tumor-reactive CD8^+^ CTLs by this combination regimen also have important implications for other cancer immunotherapies, including immune checkpoint blockade.

## Data availability statement

The raw data supporting the conclusions of this article will be made available by the authors, without undue reservation.

## Ethics statement

The animal study was reviewed and approved by IACUC of Virginia Commonwealth University.

## Author contributions

CG, X-YW designed and supervised research. JQ, XY, ZL, JC and CG performed experiments and collected data. JQ, XY, MM, HY, CG and X-YW analyzed data. CG and X-YW wrote the manuscript. All authors contributed to the article and approved the submitted version.

## References

[1] Srivastava P. Interaction of heat shock proteins with peptides and antigen presenting cells: chaperoning of the innate and adaptive immune responses. Annu Rev Immunol (2002) 20:395–425. doi: 10.1146/annurev.immunol.20.100301.064801 11861608

[2] Wang XY Facciponte JG Subjeck JR. Molecular chaperones and cancer immunotherapy. Handb Exp Pharmacol (2006) 172(172):305–29. doi: 10.1007/3-540-29717-0_13 16610365

[3] Guo C Manjili MH Subjeck JR Sarkar D Fisher PB Wang XY. Therapeutic cancer vaccines: past, present, and future. Adv Cancer Res (2013) 119:421–75. doi: 10.1016/B978-0-12-407190-2.00007-1 PMC3721379 23870514

[4] Manjili MH Henderson R Wang XY Chen X Li Y Repasky E. Development of a recombinant HSP110-HER-2/neu vaccine using the chaperoning properties of HSP110. Cancer Res (2002) 62(6):1737–42. 11912148

[5] Wang XY Chen X Manjili MH Repasky E Henderson R Subjeck JR. Targeted immunotherapy using reconstituted chaperone complexes of heat shock protein 110 and melanoma-associated antigen gp100. Cancer Res (2003) 63(10):2553–60. 12750279

[6] Manjili MH Wang XY Chen X Martin T Repasky EA Henderson R. HSP110-HER2/neu chaperone complex vaccine induces protective immunity against spontaneous mammary tumors in HER-2/neu transgenic mice. J Immunol (2003) 171(8):4054–61. doi: 10.4049/jimmunol.171.8.4054 14530326

[7] Park J Facciponte JG Chen X MacDonald IJ Repasky E Manjili MH. Chaperoning function of stress protein grp170, a member of the hsp70 superfamily, is responsible for its immunoadjuvant activity. Cancer Res (2006) 66(2):1161–8. doi: 10.1158/0008-5472.CAN-05-2609 16424054

[8] Wang XY Sun X Chen X Facciponte J Repasky EA Kane J. Superior antitumor response induced by large stress protein chaperoned protein antigen compared with peptide antigen. J Immunol (2010) 184(11):6309–19. doi: 10.4049/jimmunol.0903891 PMC3024709 20439916

[9] Wang H Yu X Guo C Zuo D Fisher PB Subjeck JR. Enhanced endoplasmic reticulum entry of tumor antigen is crucial for cross-presentation induced by dendritic cell-targeted vaccination. J Immunol (2013) 191(12):6010–21. doi: 10.4049/jimmunol.1302312 PMC3858385 24218449

[10] Murshid A Gong J Calderwood SK. Heat-shock proteins in cancer vaccines: agents of antigen cross-presentation. Expert Rev Vaccines (2008) 7(7):1019–30. doi: 10.1586/14760584.7.7.1019 18767951

[11] Wach MM Subjeck JR Wang XY Repasky E Matsuzaki J Yu H. Recombinant human Hsp110-gp100 chaperone complex vaccine is nontoxic and induces response in advanced stage melanoma patients. Melanoma Res (2022) 32(2):88–97. doi: 10.1097/CMR.0000000000000796 35254331 PMC8985419

[12] Platt N Gordon S. Is the class a macrophage scavenger receptor (SR-a) multifunctional? - the mouse's tale. J Clin Invest (2001) 108(5):649–54. doi: 10.1172/JCI200113903 PMC209390 11544267

[13] Yu X Zuo D Subjeck JR Wang XY. Scavenger receptor a (SRA/CD204): a multifaceted regulator of inflammatory response and immunity. In: Manjili MH, editor. Cytokines: Mechanisms, functions and abnormalities. (New York: Nova Biomedical) (2012).

[14] Suzuki H Kurihara Y Takeya M Kamada N Kataoka M Jishage K. A role for macrophage scavenger receptors in atherosclerosis and susceptibility to infection. Nature (1997) 386(6622):292–6. doi: 10.1038/386292a0 9069289

[15] Ricci R Sumara G Sumara I Rozenberg I Kurrer M Akhmedov A. Requirement of JNK2 for scavenger receptor a-mediated foam cell formation in atherogenesis. Science (2004) 306(5701):1558–61. doi: 10.1126/science.1101909 15567863

[16] Wang XY Facciponte J Chen X Subjeck JR Repasky EA. Scavenger receptor-a negatively regulates antitumor immunity. Cancer Res (2007) 67(10):4996–5002. doi: 10.1158/0008-5472.CAN-06-3138 17510431

[17] Yi H Yu X Gao P Wang Y Baek SH Chen X. Pattern recognition scavenger receptor SRA/CD204 down-regulates toll-like receptor 4 signaling-dependent CD8 T-cell activation. Blood (2009) 113(23):5819–28. doi: 10.1182/blood-2008-11-190033 PMC2700321 19349620

[18] Guo C Yi H Yu X Zuo D Qian J Yang G. In situ vaccination with CD204 gene-silenced dendritic cell, not unmodified dendritic cell, enhances radiation therapy of prostate cancer. Mol Cancer Ther (2012) 11(11):2331–41. doi: 10.1158/1535-7163.MCT-12-0164 PMC3496075 22896667

[19] Yu X Yi H Guo C Zuo D Wang Y Kim HL. Pattern pecognition scavenger receptor CD204 attenuates toll-like receptor 4-induced NF-{kappa}B activation by directly inhibiting ubiquitination of tumor necrosis factor (TNF) receptor-associated factor 6. J Biol Chem (2011) 286(21):18795–806. doi: 10.1074/jbc.M111.224345 PMC3099696 21460221

[20] Yi H Guo C Yu X Gao P Qian J Zuo D. Targeting the immunoregulator SRA/CD204 potentiates specific dendritic cell vaccine-induced T-cell response and antitumor immunity. Cancer Res (2011) 71(21):6611–20. doi: 10.1158/0008-5472.CAN-11-1801 PMC3213980 21914786

[21] Guo C Yi H Yu X Hu F Zuo D Subjeck JR. Absence of scavenger receptor a promotes dendritic cell-mediated cross-presentation of cell-associated antigen and antitumor immune response. Immunol Cell Biol (2012) 90(1):101–8. doi: 10.1038/icb.2011.10 PMC3134534 21383767

[22] Yu X Guo C Fisher PB Subjeck JR Wang XY. Scavenger receptors: Emerging roles in cancer biology and immunology. Adv Cancer Res (2015) 128:309–64. doi: 10.1016/bs.acr.2015.04.004 PMC4631385 26216637

[23] Martinez FO Gordon S Locati M Mantovani A. Transcriptional profiling of the human monocyte-to-Macrophage differentiation and polarization: New molecules and patterns of gene expression. J Immunol (2006) 177(10):7303–11. doi: 10.4049/jimmunol.177.10.7303 17082649

[24] Komohara Y Takemura K Lei XF Sakashita N Harada M Suzuki H. Delayed growth of EL4 lymphoma in SR-a-deficient mice is due to upregulation of nitric oxide and interferon-γ production by tumor-associated macrophages. Cancer Sci (2009) 100(11):2160–6. doi: 10.1111/j.1349-7006.2009.01296.x PMC11158051 19694752

[25] Qian J Yi H Guo C Yu X Zuo D Chen X. CD204 suppresses large heat shock protein-facilitated priming of tumor antigen gp100-specific T cells and chaperone vaccine activity against mouse melanoma. J Immunol (2011) 187(6):2905–14. doi: 10.4049/jimmunol.1100703 PMC3169704 21832164

[26] Mao S Sun W Kissel T. Chitosan-based formulations for delivery of DNA and siRNA. Adv Drug Delivery Rev (2010) 62(1):12–27. doi: 10.1016/j.addr.2009.08.004 19796660

[27] Ragelle H Vandermeulen G Préat V. Chitosan-based siRNA delivery systems. J Control Release (2013) 172(1):207–18. doi: 10.1016/j.jconrel.2013.08.005 23965281

[28] Overwijk WW Theoret MR Finkelstein SE Surman DR de Jong LA Vyth-Dreese FA. Tumor regression and autoimmunity after reversal of a functionally tolerant state of self-reactive CD8+ T cells. J Exp Med (2003) 198(4):569–80. doi: 10.1084/jem.20030590 PMC2194177 12925674

[29] Guo C Subjeck JR Wang XY. Creation of recombinant chaperone vaccine using Large heat shock protein for antigen-targeted cancer immunotherapy. Methods Mol Biol (2018) 1709:345–57. doi: 10.1007/978-1-4939-7477-1_25 PMC5812279 29177671

[30] Andersen M Howard KA Kjems J. RNAi using a chitosan/siRNA nanoparticle system: *in vitro* and *in vivo* applications. Methods Mol Biol (2009) 555:77–86. doi: 10.1007/978-1-60327-295-7_6 19495689

[31] Howard KA Paludan SR Behlke MA Besenbacher F Deleuran B Kjems J. Chitosan/siRNA nanoparticle-mediated TNF-alpha knockdown in peritoneal macrophages for anti-inflammatory treatment in a murine arthritis model. Mol Ther (2009) 17(1):162–8. doi: 10.1038/mt.2008.220 PMC2834976 18827803

[32] Morales JK Kmieciak M Graham L Feldmesser M Bear HD Manjili MH. Adoptive transfer of HER2/neu-specific T cells expanded with alternating gamma chain cytokines mediate tumor regression when combined with the depletion of myeloid-derived suppressor cells. Cancer Immunol Immunother (2009) 58(6):941–53. doi: 10.1007/s00262-008-0609-z PMC3083860 18979098

[33] Yu X Liu W Chen S Cheng X Paez PA Sun T. Immunologically programming the tumor microenvironment induces the pattern recognition receptor NLRC4-dependent antitumor immunity. J Immunother Cancer (2021) 9(1):e001595. doi: 10.1136/jitc-2020-001595 33468554 PMC7817794

[34] Quah BJ Parish CR. The use of carboxyfluorescein diacetate succinimidyl ester (CFSE) to monitor lymphocyte proliferation. J Vis Exp (2010) 44. doi: 10.3791/2259 PMC3185625 20972413

[35] Koppolu B Zaharoff DA. The effect of antigen encapsulation in chitosan particles on uptake, activation and presentation by antigen presenting cells. Biomaterials (2013) 34(9):2359–69. doi: 10.1016/j.biomaterials.2012.11.066 PMC3552013 23274070

