# Supplementary material for: SRA inhibition improves antitumor potency of antigen-targeted chaperone vaccine
Source: Front Immunol. 2023 Jan 30;14:1118781. doi: 10.3389/fimmu.2023.1118781 (PMC9923017; doi:10.3389/fimmu.2023.1118781)
Supplement: Supplementary file 1 [file Presentation_1.pptx]

## Slide 1
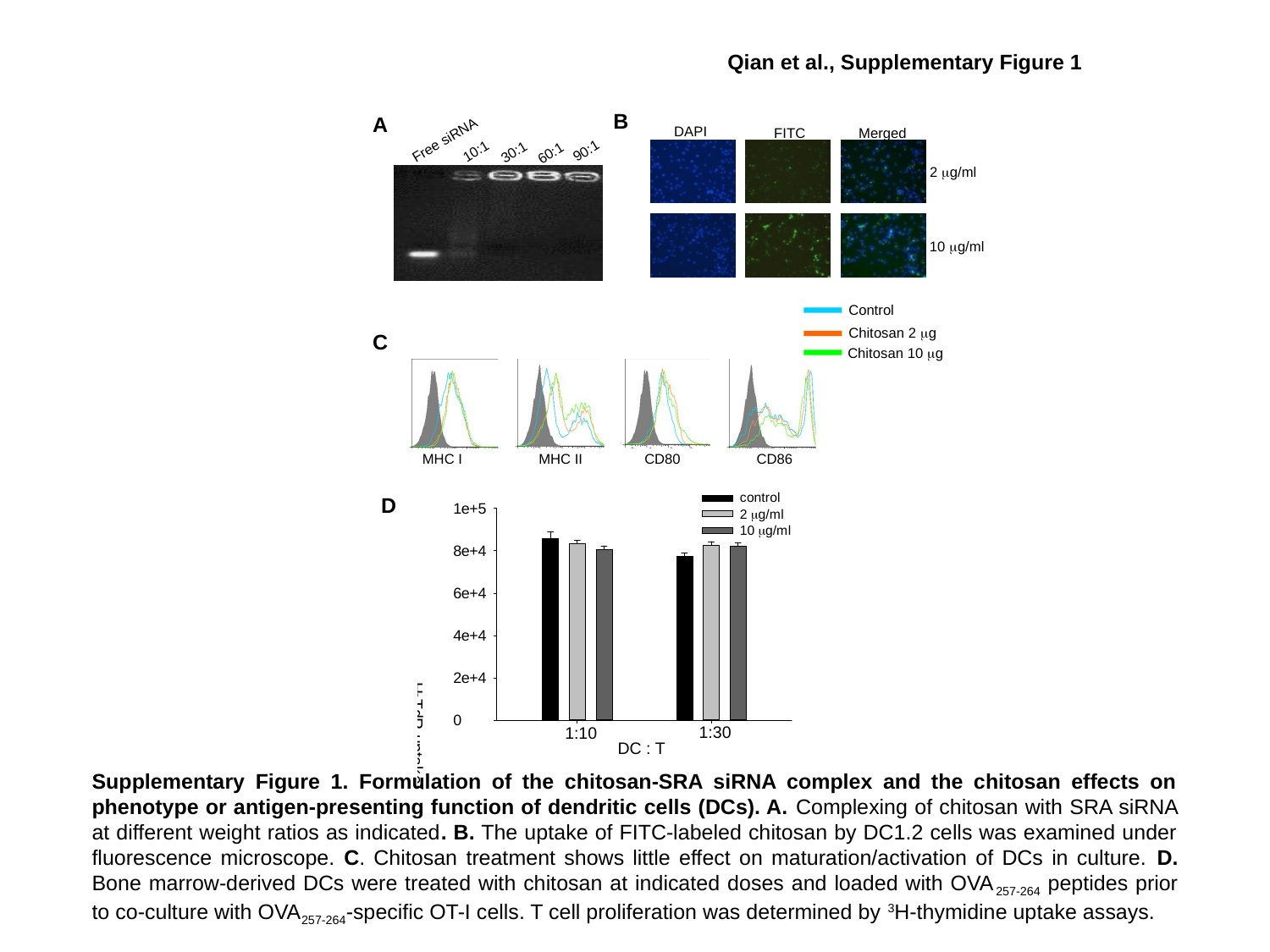

Qian et al., Supplementary Figure 1
B
A
Free siRNA
DAPI
Merged
FITC
2 μg/ml
10 μg/ml
90:1
10:1
30:1
60:1
Control
Chitosan 2 μg
Chitosan 10 μg
C
MHC I
MHC II
CD86
CD80
D
Supplementary Figure 1. Formulation of the chitosan-SRA siRNA complex and the chitosan effects on phenotype or antigen-presenting function of dendritic cells (DCs). A. Complexing of chitosan with SRA siRNA at different weight ratios as indicated. B. The uptake of FITC-labeled chitosan by DC1.2 cells was examined under fluorescence microscope. C. Chitosan treatment shows little effect on maturation/activation of DCs in culture. D. Bone marrow-derived DCs were treated with chitosan at indicated doses and loaded with OVA257-264 peptides prior to co-culture with OVA257-264-specific OT-I cells. T cell proliferation was determined by 3H-thymidine uptake assays.

## Slide 2
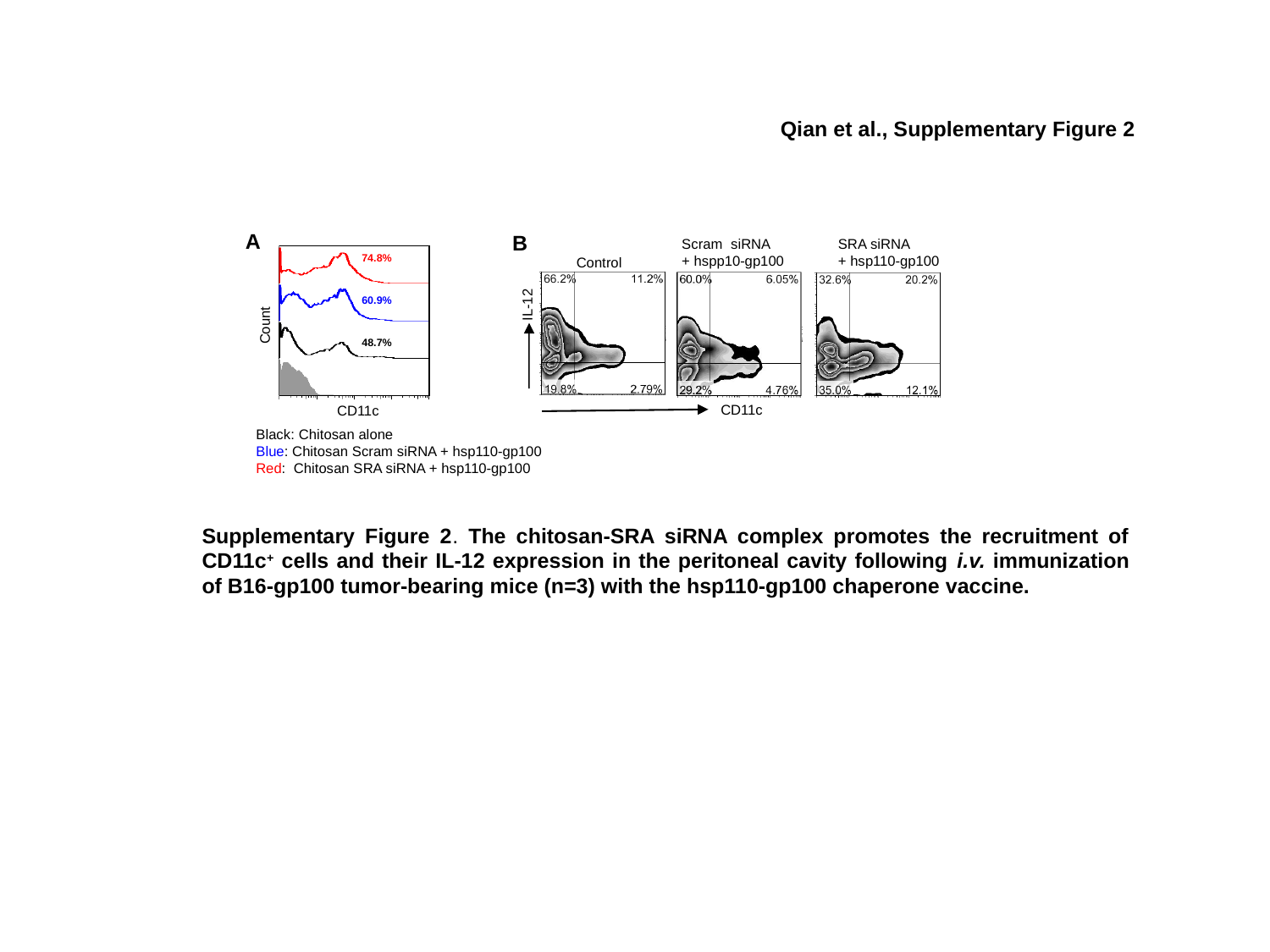

Qian et al., Supplementary Figure 2
A
74.8%
60.9%
Count
48.7%
CD11c
Black: Chitosan alone
Blue: Chitosan Scram siRNA + hsp110-gp100
Red: Chitosan SRA siRNA + hsp110-gp100
B
Scram siRNA
+ hspp10-gp100
SRA siRNA
+ hsp110-gp100
Control
IL-12
CD11c
Supplementary Figure 2. The chitosan-SRA siRNA complex promotes the recruitment of CD11c+ cells and their IL-12 expression in the peritoneal cavity following i.v. immunization of B16-gp100 tumor-bearing mice (n=3) with the hsp110-gp100 chaperone vaccine.

## Slide 3
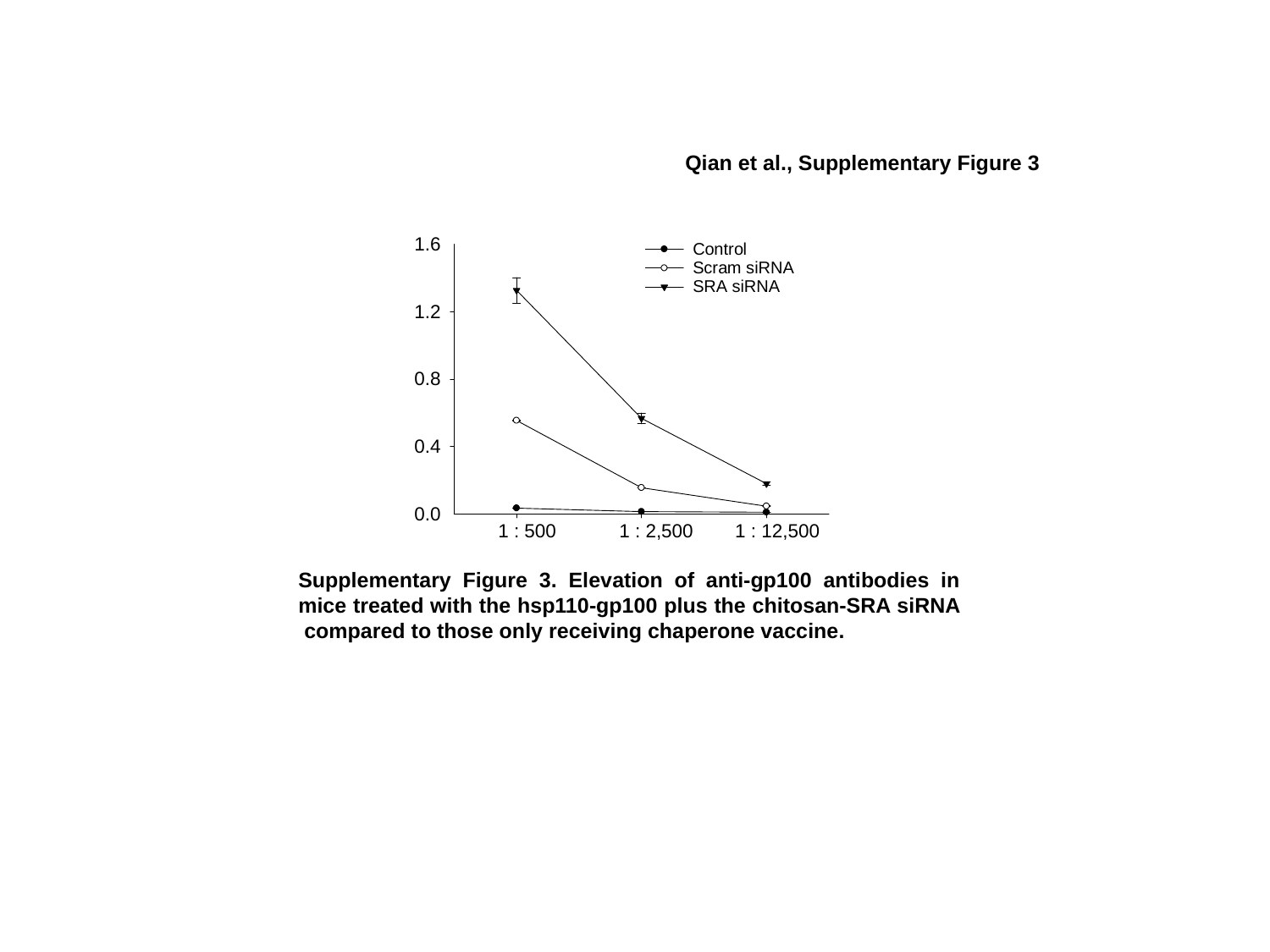

Qian et al., Supplementary Figure 3
Supplementary Figure 3. Elevation of anti-gp100 antibodies in mice treated with the hsp110-gp100 plus the chitosan-SRA siRNA compared to those only receiving chaperone vaccine.

## Slide 4
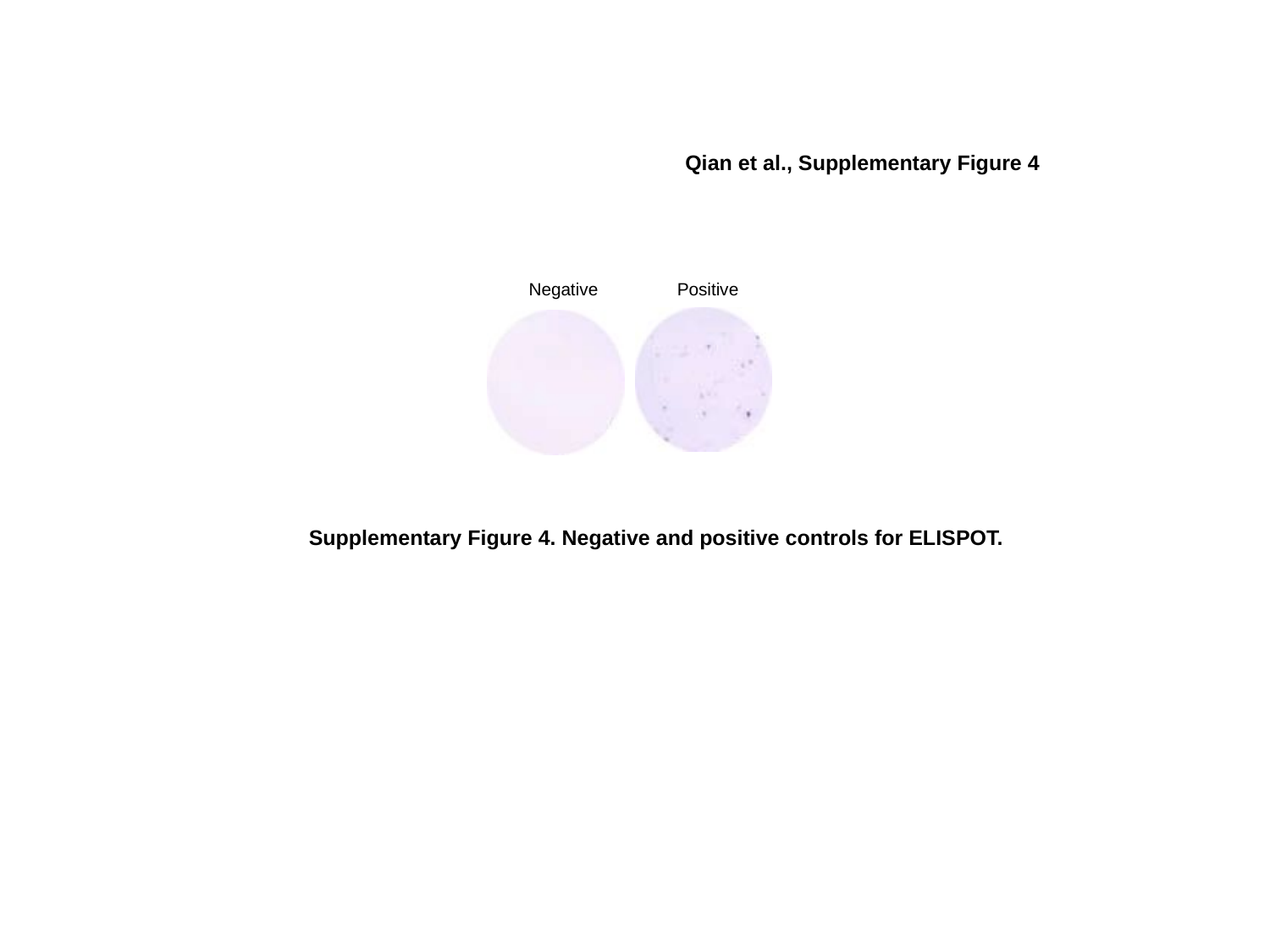

Qian et al., Supplementary Figure 4
Negative
Positive
Supplementary Figure 4. Negative and positive controls for ELISPOT.
